# Vein of Galen Aneurysmal Malformation Presenting As Persistent Pulmonary Hypertension in a Newborn: A Case Report

**DOI:** 10.7759/cureus.67191

**Published:** 2024-08-19

**Authors:** Barbara Amendolia, Vishwanath Bhat, Erica Poletto, Amar Jaspreet, Bradley Robinson

**Affiliations:** 1 Neonatal Intensive Care Medicine, Stockton University, Galloway, USA; 2 Neonatal Intensive Care Medicine, Cooper University Hospital, Camden, USA; 3 Radiology, Cooper University Hospital, Camden, USA; 4 Cardiology, Nemours Children's Hospital, Philadelphia, USA

**Keywords:** neonatal neurology, case report, persistent pulmonary hypertension of the newborn, vein of galen, arteriovenous malformation

## Abstract

We present a case of a newborn infant with a vein of Galen aneurysmal malformation (VOGM) who was admitted to our neonatal intensive care unit (NICU) with the diagnosis of persistent pulmonary hypertension of the newborn (PPHN). Further work-up at our institution, which included an echocardiogram and cranial ultrasound revealed VOGM. The patient was transferred to a children’s center for further management of the vascular malformation where the patient subsequently died from high cardiac output heart failure. This study highlights the importance of considering a VOGM as a rare cause of PPHN in an infant.

## Introduction

The vein of Galen arteriovenous malformation is a very rare malformation that accounts for 30% of pediatric congenital vascular malformations and 1% of all pediatric congenital anomalies [1]. While the true incidence is unknown, it is estimated to be about one in every 10,000 to 25,000 births [2]. Diagnosis may be made prenatally, during the neonatal period, or even later in childhood. The clinical presentation can be very different depending on the timing and onset of symptoms/diagnosis [3]. Although the classic clinical feature in neonates is heart failure, neonatal providers do not often consider the diagnosis of vein of Galen aneurysmal malformation (VOGM) in a sick newborn presenting with persistent pulmonary hypertension. A family history of hereditary hemorrhagic telangiectasia (Osler-Weber-Rendu syndrome) has been noted in some cases [4]. This syndrome was not present in our patient.

## Case presentation

The patient is a newborn male delivered via stat cesarean section to a G3P0111 mother at 41+2/7 weeks gestation for breech presentation and maternal hypertension. Maternal history is significant for lack of prenatal care and polysubstance use. Rupture of membranes occurred at delivery and the amniotic fluid was meconium stained. The Apgar scores were 1, 6, and 8 at 1, 5, and 10 minutes, respectively. In the delivery room, he required positive pressure ventilation (PPV) briefly and was transitioned to continuous positive airway pressure (CPAP). An initial sepsis workup was performed but antibiotics were discontinued after 36 hours with negative blood culture results. He required phototherapy for one day for hyperbilirubinemia. The patient was also being treated for neonatal abstinence syndrome due to intrauterine drug exposure to fentanyl and amphetamines. He was tolerating full enteral feedings. At the outside hospital (OSH), the patient was noted to have increasing tachypnea requiring increasing CPAP and FiO_2_. The initial echocardiogram at the OSH demonstrated severe pulmonary hypertension, suprasystemic pulmonary arterial pressure (PAP), dilatation of right atrium (RA), right ventricle (RV), and pulmonary arteries (PA) with bidirectional flow across atrial septum and patent ductus arteriosus (PDA).

He was transferred to our hospital on the day of life (DOL) #11 with worsening respiratory distress secondary to sepsis versus PPHN. Auscultation of the baby’s head did not reveal a bruit. Vital sign assessment revealed a BP of 90/36 on DOL #12 despite the ductus arteriosus being closed. He was noted to have a harsh murmur described as holosystolic heard best at the left upper sternal border. This was believed to be from increased cardiac output across his pulmonary valve. He was managed on CPAP and started on inhaled nitric oxide (iNO) 20 parts per million (PPM). An echocardiogram was performed at the OSH and a follow-up was performed at our center.

**Figure 1:**
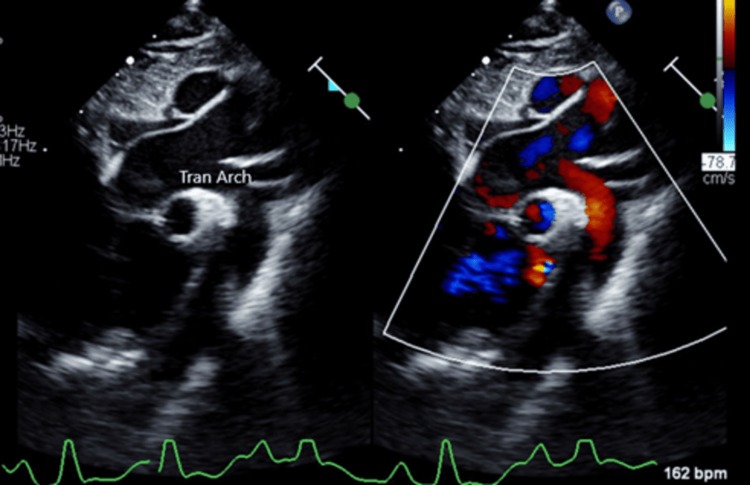
Reversal of flow in the transverse arch is consistent with a VOGM (suprasternal notch view). Normally, there is absent (red) flow in the transverse arch during diastole; however, in this case, the large runoff from the VOGM caused the reversal of flow (as indicated by the red color in the transverse arch). VOGM: vein of Galen aneurysmal malformation

**Figure 2:**
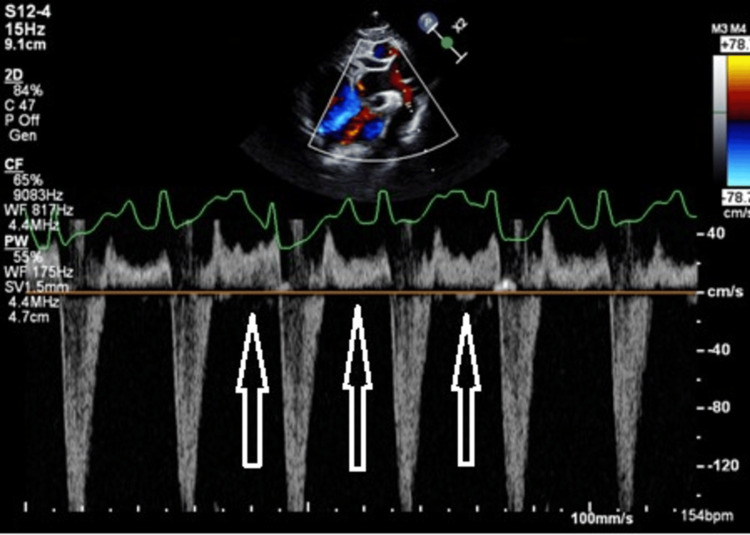
Pulse wave Doppler in the transverse aortic arch. There is unexplained diastolic flow reversal in the absence of a PDA which raised suspicion of VOGM. Diastole and systole are labeled (arrows). PDA: patent ductus arteriosus; VGAM: vein of Galen aneurysmal malformation

A repeat echocardiogram on DOL 12 revealed stretched patent foramen ovale with bidirectional flow, moderate tricuspid regurgitation with a peak gradient of 77 mmHg, blood pressure (BP) 90/36 mmHg, moderate dilation of the right atrium and right ventricle with mild dilation of the main pulmonary artery, mild pulmonary insufficiency, septal flattening of the left ventricle in systole and diastole, normal biventricular function, and closed ductal artery. Diastolic flow reversal was noted in the descending aorta back into the transverse aortic arch into the cervical neck vessels in the absence of a PDA (Figures 1, 2). There was no aortic regurgitation and an aortopulmonary (AP) window was not seen (which can also cause diastolic flow reversal) in the transverse aortic arch.

**Figure 3:**
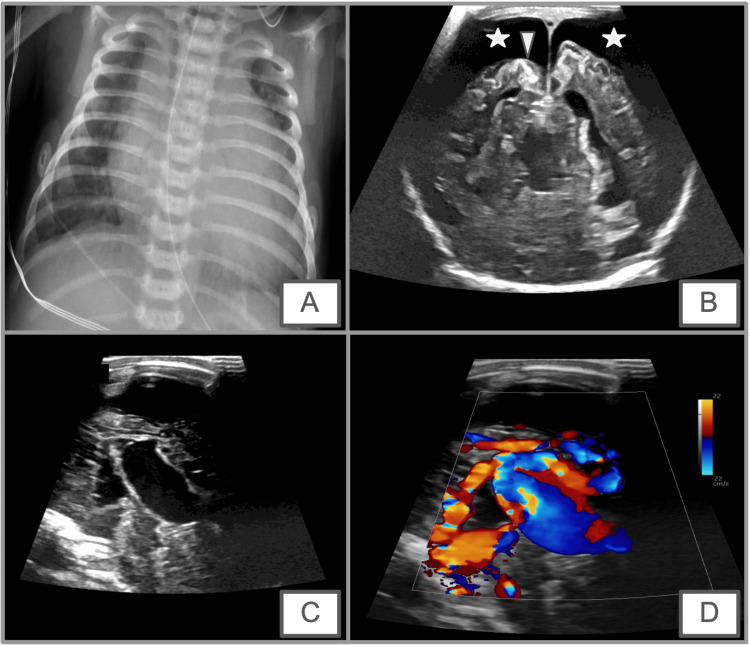
A frontal chest radiograph, brain ultrasound, midline sagittal grayscale, and color Doppler images. (A) A frontal chest radiograph obtained on DOL #11 shows a significant enlargement of the cardiac silhouette and mild enlargement of central pulmonary vasculature, which may be related to extra-cardiac shunting. (B) Coronal grayscale image from brain ultrasound obtained on DOL# 14 shows a significant parenchymal volume loss with enlargement of the extra-axial space (star). (C and D) Increased echogenicity of the frontal cortex (arrowhead) suggests laminar necrosis in the setting of prior infarction. Midline sagittal grayscale (C) and color Doppler (D). DOL: day of life

A Doppler of each pulmonary vein was performed and pulmonary vein stenosis was ruled out. A brain ultrasound to assess an arteriovenous malformation was suggested. The initial head ultrasound at the OSH was reported as normal. A repeat head ultrasound on DOL #15 revealed VOGM, suspected diffuse volume loss, with ventricular dilation and subdural fluid collections, abnormal hyperechogenicity of the cortex that was suspicious for laminar necrosis in the setting of prior/chronic ischemia and dependent intraventricular hemorrhage within the lateral ventricles (Figures 3A-3D).

A frontal chest radiograph obtained on DOL #11 revealed significant enlargement of the cardiac silhouette and mild enlargement of central pulmonary vasculature most likely related to the large left to right shunting (Figures 3A-3D).

The infant was transferred to a tertiary children’s hospital for further evaluation and management. After evaluation from multiple sub-specialties including radiology, neurosurgery, neurology, and cardiology, the consensus was that intervention was unlikely to improve the outcome of the extensive brain lesion. Patients with abnormal brain anatomy, tricuspid regurgitation, and large VOGM typically have worse outcomes [1]. Our patient had a large VOGM lesion, tricuspid regurgitation, and associated brain finding. For this reason, endovascular intervention to close the malformation was not recommended. Parents were encouraged to provide care and support for the baby as they were able, without escalating therapy or support further. The infant died on DOL #44 from high-output heart failure.

## Discussion

With improving ultrasound technology, VOGM is increasingly being detected in routine antenatal ultrasounds. The typical appearance is a midline anechoic structure posterior to the third ventricle on grayscale imaging, with turbulent arterial and venous flow. Fetal ultrasound may also detect associated cerebral abnormalities, which include hydrocephalus and encephalomalacia. Fetal ultrasound can be used to evaluate cardiac function and stigmata of cardiac failure, which can include cardiomegaly, tricuspid insufficiency, polyhydramnios, pericardial/pleural effusion, ascites, and subcutaneous edema [4]. When suspected, fetal brain MRI may be useful in confirming the diagnosis, and evaluating for concurrent cerebral abnormalities, as well as signs of hydrops due to cardiac failure [5].

If not detected antenatally, VOGM may be incidentally detected on neurosonography performed for other reasons, or if the patient undergoes ultrasound for findings associated with the hydrocephalus or encephalomalacia, which can occur in these patients. Such signs and symptoms can include macrocephaly, developmental delay, prominent scalp veins, or seizure [6]. Postnatal ultrasound findings are similar to antenatal ultrasound findings.

Alternatively, the diagnosis may be suspected in a patient with unexplained signs and symptoms of heart failure. Chest radiography is often performed in such patients prior to neurosonography. Cardiomegaly from high-output heart failure is the most common radiographic finding, as in our patient, while pulmonary edema and pleural effusion may also be seen.

In patients who are not present in the neonatal period, symptoms related to venous hypertension may be present, including headache, seizure, and focal neurologic deficits [7]. VOGM may be detected on head CT or MRI in these patients.

The proposed mechanism by which pulmonary hypertension develops in infants with VOGM involves the left-to-right shunt due to the low resistance brain malformation and subsequent volume overload on the right side of the heart and the lungs. This likely occurs in utero as the increased volume to the right side of the heart and pulmonary artery is too much for the PDA to carry (relative PDA stenosis) and thus goes to the lungs resulting in pulmonary artery hypertension [8-10].

On imaging, there are following two types of malformations, as classified by Lasjaunias: choroidal and mural [4]. The choroidal type has multiple feeding vessels draining to the anterior aspect of the median prosencephalic vein. This is the more common type of malformation, and unfortunately is more complex to treat and is associated with worse clinical outcomes and survival rates. The mural type has single or multiple fistulas of the median prosencephalic vein and may present later in infancy, childhood, or rarely adulthood [4].

Catheter angiography can be used for precise delineation of the vascular anatomy, as well as treatment with embolization. Embolization can be achieved with liquid embolization, coils, or a combination of these strategies [11]. Many separate embolization procedures may be required for complete occlusion of the malformation. Embolization was not considered appropriate for our patient because of the extensive size of the VOGM.

## Conclusions

It is often difficult to determine the etiology of PPHN in a neonate. This study highlights the need for providers to consider VOGM in the presence of PPHN. These patients usually present with right-sided volume overload and cardiomegaly. From an ultrasound diagnostic standpoint, one must always explain the reversal of diastolic flow in the transverse aortic arch in the absence of a PDA. Consideration of VOGM in a newborn with PPHN may help with early diagnosis which can in turn guide management strategies and improve outcomes.
